# Social Media as a Form of Virtual Whistleblowing: Empirical Evidence for Elements of the Diamond Model

**DOI:** 10.1007/s10551-020-04598-y

**Published:** 2020-08-17

**Authors:** Hengky Latan, Charbel Jose Chiappetta Jabbour, Ana Beatriz Lopes de Sousa Jabbour

**Affiliations:** 1 HLC Consulting, Jl. Kertanegara Selatan V No 5B, Semarang, 50241 Indonesia; 2 Lincoln International Business School, University of Lincoln, Lincoln, LN6 7TS Lincolnshire UK; 3 Montpellier Business School, 2300, avenue des Moulins, 34185 Montpellier, Cédex4, France

**Keywords:** Business ethics, Online whistleblowing, Pressure, Financial incentive, Opportunity, Rationalization, Capability

## Abstract

This article originally advances the field of organizational whistleblowing by empirically investigating the suitability of the four elements of the fraud diamond as a means to understand the intention to disclose wrongdoing through virtual channels. This article also makes a contribution on the theme of whistleblowing as it relates to customers, an under-studied, however, relevant stakeholder in this field. The main findings of the article are as follows: (a) the four elements of the fraud diamond as they relate to whistleblowing—a combination of pressure, financial incentive, opportunity and rationalization, and capability—can explain the intentions behind customer reports of wrongdoing; (b) online social media channels are customers’ preferred means of whistleblowing; (c) the elements of opportunity and capability are strongly correlated with the use of social media as a method of disclosing wrongdoing; and (d) virtual channels can be useful for whistleblowers in order to avoid potential retaliation. Unique managerial and academic implications of these research findings are also discussed, extending the layers of knowledge on whistleblowing in organizations.

## Introduction

In recent years, the total global economic loss incurred due to fraud and wrongdoing in organizations has increased. A study conducted by the Association of Certified Fraud Examiners (ACFE) in 2016 reported that this total loss exceeded $6.3 billion, rising to $7 billion in 2018 (ACFE 2018). Parallelly, the number of whistleblowers who have observed and reported such wrongdoing has also increased. This group is dominated by employees (53%), followed by customers (21%) and then by anonymous/other whistleblowers (ACFE 2018). Whistleblowers have also played an important role during the 2020 coronavirus pandemic (Brown 2020).

Whistleblowers play a vital role in revealing wrongdoing in contemporary organizations (Andrade 2015; Mason and Simmons 2018; Miceli and Near 2002; Loyens and Maesschalck 2014), which are considerably more complex and influenced by digital technologies; and context has unleashed a shift in how these wrongdoings tend to be exposed (Bosua et al. 2014; Lam and Harcourt 2019; Munro 2017). As Vandekerckhove et al. (2014a) outline, the use of online channels or social media can be considered a contemporary approach to exposing wrongdoing. This paper refers to the use of such methods as ‘virtual’ or ‘online’ whistleblowing (Cherry 2012; Lam and Harcourt 2019). Previous studies have linked whistleblowers’ use of both internal channels, which involve reporting within the organization, for example to a supervisor, and external channels, which involve reporting outside the organization, for example to a news or government agency (Alleyne et al. 2018; Latan et al. 2018; Lee and Fargher 2018; Park and Blenkinsopp 2009; Robertson et al. 2011; Skivenes and Trygstad 2010). However, there has been an acute lack of discussion and significant lack of empirical evidence concerning virtual whistleblowing in general.

To date, no existing research has considered virtual whistleblowing channels as a contemporary approach to reporting wrongdoing in a context of acute digitalization of contemporary organizations. Sharing information and speaking out about wrongdoing has become easier with the rapid proliferation of information technology, allowing individuals or groups to socialize and connect online across time and space. As pointed out by Bosua et al. (2014), these aspects of social media and online platforms have had a significant impact on potential whistleblowers. In addition, other benefits of online channels may be taken into account by whistleblowers, including speed of communication or sharing information, range of options, anonymity, ease of use and cost.

As a corollary of this debate, the main objective of this work is to empirically test the factors that influence whistleblowers in revealing wrongdoing through virtual whistleblowing channels. Specifically, this work integrates the recently developed concept of the whistleblowing triangle (Latan et al. 2019c; Smaili and Arroyo 2019) with the fraud diamond perspective. The whistleblowing triangle, which is an adaptation of the prior concept of the fraud triangle (Dellaportas 2013; Free 2015), is composed of three sides, each comprised of one or more elements, used to understand the intention behind the reporting of wrongdoing (Latan et al. 2019c; Smaili and Arroyo 2019; Wolfe and Hermanson 2004), namely: (i) pressure (PRS) or financial incentive (FNI); (ii) opportunity (OPR); and (iii) rationalization (RNL). The fraud diamond perspective proposes the addition of a fourth element into the fraud triangle concept, which is the capability (CPB) of the whistleblower. Wolfe and Hermanson (2004) argue that this fourth element of the fraud diamond—capability—should be considered in analyses of the factors that lead people to report wrongdoing, because capability empowers individuals to turn an opportunity to disclose wrongdoing into reality.

As far as we are aware, only a few previous studies have tested the components of the whistleblowing triangle (Andon et al. 2018; Brown et al. 2016; Latan et al. 2019c; MacGregor and Stuebs 2014), and this is still considered a research gap. As proposed by Wolfe and Hermanson (2004), the fourth element of the fraud diamond—capability—which is absent in the original fraud triangle model, must be added to the fraud triangle concept, as well as to the whistleblowing triangle (Latan et al. 2019c; Smaili and Arroyo 2019). Consequently, this research considers this fourth ‘diamond element’ in order to understand the motivations behind virtual whistleblowing intention.

According to Smaili and Arroyo (2019) and Latan et al. (2019c), there are two types of pressure: positive (internal pressure) and negative (external pressure). This study focuses on internal pressure, which positively encourages whistleblowing. This type of pressure relates to whistleblowers’ personal moral and religious values and sense of social duty, and therefore comes from within. Financial incentives can also motivate observers to speak out about wrongdoing (Andon et al. 2018; Rose et al. 2018; Friebel and Guriev 2012), which relates to whistleblowers’ expectations (Berger et al. 2017; Brown et al. 2016; Lee and Turner 2017). The financial incentives available to whistleblowers differ between nations, and depend on the relevant local legal regulations, such as the Dodd-Frank legislation in the US; in Indonesia, such financial incentives do exist, but are not explicitly mentioned or legislated.

Furthermore, observers will often choose the easiest opportunity (e.g., means and channel) to blow the whistle, taking into account future risks and potential retaliation (Guthrie and Taylor 2017; Kaplan et al. 2012; Rehg et al. 2008). In some cases, the complexity of using certain channels may deter whistleblowers from revealing wrongdoing (Casal and Bogui 2008; MacGregor and Stuebs 2014). Conversely, online channels, such as WikiLeaks, can ensure anonymity, while the sharing of information tends to be limited to certain groups on channels such as Facebook or Twitter and other social media sites. However, in many cases, individuals go through a process of rationalization before deciding whether to blow the whistle or remain silent when faced with wrongdoing, before helping the victims of fraud (Brown et al. 2016; Latan et al. 2019b; Smaili and Arroyo 2019). The use of online channels allows whistleblowers to share information about wrongdoing quickly and widely, minimizing the potential for harm to victims. Finally, whistleblowers’ ability, confidence and skills help them in revealing wrongdoing through online channels.

Therefore, this study aims to test a virtual whistleblowing model, providing the first empirical evidence on this topic using a research sample of Indonesian customers. As pointed out by Culiberg and Mihelič (2017), customers have received little attention in the whistleblowing literature to date, with most previous studies using organizational members as the research sample (e.g., employees, managers, internal auditors, audit committees etc.). However, external whistleblowers, including customers, can also be considered whistleblowers when they observe misconduct through direct interaction with the organization. This process is in reality no different from members of an organization identifying wrongdoing—it differs only in the way in which wrongdoing is observed and discovered. Given that food fraud and wrongful business practices have recently increased (Moy 2018), this perspective allows customers to engage in blowing the whistle. This paper argues that customers often observe wrongdoing by organizations, and that they should therefore be seen as active subjects in the area of whistleblowing (ACFE 2018). Furthermore, our model is tested in Indonesia; most extant research on this subject is based in Western countries, while studies in developing countries are relatively rare (Alleyne et al. 2017; Latan et al. 2018; Miceli and Near 2005). More importantly, Indonesia has the fourth largest population in the world, after China, India and the U.S, and is among the world’s most enthusiastic nations in terms of internet use. For all the above reasons, it is undoubtedly worth testing this virtual whistleblowing model in an Indonesian context.

This study both broadens and deepens our understanding of the field of whistleblowing, providing original evidence in three important ways. First, it responds to research suggestions from experts in the field—e.g., Vandekerckhove et al. (2014a) and Lam and Harcourt (2019) —and provides empirical evidence concerning the virtual whistleblowing model as a contemporary approach to uncovering wrongdoing. This is thought to be the first empirical study to consider online channels in relation to whistleblowing intention.

Second, this study expands the concept of the whistleblowing triangle (Smaili and Arroyo 2019; Latan et al. 2019c) by adding a fourth element—whistleblowers’ capability—creating a single comprehensive model. To date, there has been a lack of empirical evidence relating to this concept in the whistleblowing literature, which is considered a persistent research gap. As far as we are aware, this is the first empirical study to apply the four elements of the fraud diamond to predicting whistleblowing intention (Latan et al. 2019c; Smaili and Arroyo 2019; Wolfe and Hermanson 2004). Finally, the use of customers as the research sample is novel. As customers are considered a unique group of ‘external whistleblowers’, operating outside the boundaries of the organization, they are free from various risks and obstacles (for example, threat of dismissal, poor performance appraisal, unfair treatment, intimidation or verbal harassment). Therefore, they are not involved in conflicts related to professional ethics and loyalty, as organizational members are (Bouville 2008; Jubb 1999; Varelius 2009). However, other risks remain and may threaten them, such as lawsuits from unethical companies or requests for compensation due to disclosure of wrongdoing.

The remainder of this paper is organized as follows. The next section presents the theoretical background and development of hypotheses, followed by the research methodology. Following this, the empirical results are presented. Finally, the results are discussed and implications for both academics and practitioners are given.

## Theoretical Background and Development of Hypotheses

### Whistleblowing as Prosocial Behavior

Whistleblowing has been defined by a number of scholars from various perspectives (Alford 2001; Dozier and Miceli 1985; Jubb 1999; King 1997; Near and Miceli 2011; Vinten 2000). One definition of whistleblowing that has been widely accepted in social science research is that whistleblowing constitutes the disclosure by members of an organization (including former members and job applicants) of illegal, immoral, or illegitimate practices (including omissions) by the employer, to persons or organizations who may be able to effect action (Near and Miceli 1985). According to this definition, only members of the organization can be considered whistleblowers. However, this paper argues that, due to advancements in digital technology, this definition is too narrow and restrictive, because access to relevant information is not always limited to members of the organization. Hence, wrongdoing is not only observed by organizational insiders, but also by outsiders such as customers, vendors, consultants, external auditors or competitors. For instance, customers who observe instances of food fraud in Indonesia can report their findings to formal agencies such as the consumer protection agency or the national agency of drug and food control, through online channels. These agencies tend to take decisive action against wrongdoing, such as removing products from the market and even withdrawing production permits.

In addition, it is important to distinguish between bell-ringers and whistleblowers, as highlighted by Miceli et al. (2014). Someone is called a bell-ringer when they suspect organizational wrongdoing and disseminate this information. In such a case, the bell-ringer does not necessarily intend to stop the wrongdoing, and has not directly observed the suspected misconduct in the workplace. Meanwhile, whistleblowers are the opposite. They observe wrongdoing directly and intend to stop it in order to help the victims. Therefore, in this paper, a broader definition of whistleblowing is adopted: whistleblowing is a deliberate, non-obligatory act of disclosure. It is made by a person who has—or has had—privileged access to an organization’s data or information concerning non-trivial illegality or other wrongdoing, whether actual, suspected or anticipated, which implicates, and is under the control of, that organization, to an external entity which has the potential to rectify that wrongdoing (Jubb 1999).

Whistleblowing is regarded as a prosocial behavior; that is, a behavior intended to benefit others, in this case by uncovering wrongdoing in an organization (Alford 2001; Latan et al. 2018; Miceli et al. 2008). As proposed by Dozier and Miceli (1985) in the Prosocial Organizational Behavior (POB) Model, whistleblowing is viewed as a prosocial behavior when the potential whistleblower observes wrongdoing and this motivates them to undertake three phases of action (Brief and Motowidlo 1986; Miceli et al. 2008). The first phase involves observing a questionable activity and labeling it as wrongful. In the second phase, the observer reacts to the wrongdoing by experiencing it as incorrect. Finally, in the third phase, the observer decides on a course of action where whistleblowing is an available option (Bjørkelo and Bye 2014; Near and Miceli 2011). Miceli et al. (2008) point out that this behavior does not have to be altruistic to be considered prosocial, and that while whistleblowers may feel morally compelled to act, they may simultaneously hold the view that the disclosure will result in some personal gain for themselves.

### The Whistleblowing Diamond

In a recent study, Smaili and Arroyo (2019) proposed a new conceptual model called the whistleblowing triangle, an adaptation of the prior concept of the fraud triangle (Dellaportas 2013; Free 2015). The whistleblowing triangle model comprises the following three sides, each comprised of one or more elements: (i) pressure (PRS) or financial incentives (FNI); (ii) opportunities (OPR); and (iii) rationalization (RNL), all of which can help explain the intention behind whistleblowing. It is worth mentioning at this juncture that the use of the term ‘triangle’ is based on the three sides of grouped factors, rather than the total number of elements in the model. However, there is a lack of understanding about the relationships between these elements, and there is little empirical evidence for the model, with only two previous studies addressing this issue. First, a study by Brown et al. (2016) uses elements of the whistleblowing triangle as a proxy to explain the use of the Theory of Planned Behavior (TPB) regarding whistleblowing intention among management accountants. Their findings indicate that attitude and perceived behavioral control have a significant effect on whistleblowing intention. Second, a study by Latan et al. (2019c) uses the original propositions of Smaili and Arroyo (2019) to test the whistleblowing triangle model. Their results show that the elements of the whistleblowing triangle work as antecedents which trigger observers to blow the whistle.

However, the triangle model, as it relates to both fraud and whistleblowing, is not the conclusive model in the business ethics literature. As Wolfe and Hermanson (2004) argue, this model can be enhanced and improved by adding a fourth element. In addition to pressure, financial incentives, opportunities and rationalization, the element of capability must be taken into account. An observer must have the capability to recognize wrongdoing and choose an open reporting channel in order to blow the whistle. The capability of the whistleblower is related to personal traits and abilities, which play a major role when revealing wrongdoing, even in the presence of other elements.

This study includes the element of capability in order to test the ‘whistleblowing diamond’ model in an Indonesian context. As pointed out by Smaili and Arroyo (2019) and Latan et al. (2019c), more comprehensive research is needed to develop the whistleblowing triangle model, and to extend it using elements of the fraud diamond model. Given the lack of empirical evidence and the limited scope of previous studies, it is vital to deepen insights in this field. The following sections will describe the components of the whistleblowing diamond and formulate hypotheses based on this model.

### Pressures Affecting Whistleblowing

Pressure has different meanings in different contexts. In this paper, pressure is defined as a positive incentive which motivates observers to reveal wrongdoing. Pressure can come from within the whistleblower (internal pressure), or outside the whistleblower (external pressure) (Latan et al. 2019c; Smaili and Arroyo 2019). Internal pressure is related to an observer’s personal moral, ethical and religious values, which may encourage him/her to uncover and reveal wrongdoing. This pressure usually arises from of a sense of social responsibility and the duty the observer feels to reveal the truth (Leys and Vandekerckhove 2014). On the other hand, external pressure relates to threats or retaliation, and can therefore be a disincentive to blow the whistle. This pressure usually reduces the whistleblower’s motivation because of its potential negative effects on career and professional life. A whistleblower usually faces external pressure when revealing serious wrongdoing (Andon et al. 2018; Latan et al. 2019b; Rehg et al. 2008; Skivenes and Trygstad 2010). Due to the research sample used in this study, external pressure may be less relevant or have little impact and, therefore, internal, positive pressure will be focused on. While external pressures such as threats of dismissal or poor performance appraisal are not relevant for external whistleblowers, these factors may be more relevant when examining a sample of individuals who are members of an organization. However, external pressures do still threaten external whistleblowers, such as lawsuits from unethical companies or requests for compensation.

In line with the Theory of Planned Behavior (TPB), a whistleblower experiences both personal and social pressure (internal), and organizational pressure (external) (Miceli et al. 2008; Smaili and Arroyo 2019). In the Indonesian context, external whistleblowers often speak out when confronted with unethical organizational behavior as a consequence of personal and social pressure, and this action is often carried out through online platforms and social media. As external whistleblowers experience less retaliation and have access to online reporting channels, they are often in a good position to reveal wrongdoing. At times, personal and social pressure may give the whistleblower greater courage, with the aim of helping victims and preventing wider damage. Conversely, internal whistleblowers often choose to remain silent about observed wrongdoing, as a result of organizational pressure (Culiberg and Mihelič 2017; Latan et al. 2019b; MacGregor and Stuebs 2014). This is due to the lack of protection for whistleblowers when revealing organizational wrongdoing through internal mechanisms. Since there is no law clearly regulating protection for whistleblowers in Indonesia, the use of these internal channels is less effective compared to online platforms. Meanwhile, previous studies indicate that pressure has a positive effect on the intention to blow the whistle (Smaili and Arroyo 2019), and internal pressure motivates the whistleblower to act (Chen and Lai 2014; Latan et al. 2019c). Based on the above discussion, our first hypothesis is:

#### H1

Pressure has a positive effect on online whistleblowing intention.

### Whistleblowing and Financial Incentives

A whistleblower may consider financial incentives when reporting organizational misconduct. This motivating factor for uncovering wrongdoing is taken very seriously (Andon et al. 2018; Rose et al. 2018). Financial incentives and compensation schemes are designed to encourage whistleblowers to report wrongdoing which may result in large financial losses. Typically, observers use anonymous online channels to report their findings, and receive predetermined rewards. The use of anonymous online channels is intended to maintain the confidentiality of personal identities, and prevent retaliation against whistleblowers. Indeed, several regulatory bodies provide financial incentives for anyone who has information about wrongdoing in an organization. This is considered an effective way of uncovering wrongdoing in organizations, allowing for corrective action. A number of recent studies indicate that compensation and financial incentives can trigger whistleblowers to act (Andon et al. 2018; Berger et al. 2017; Friebel and Guriev 2012).

In addition to financial incentives, there are also social and moral incentives (Brown et al. 2016). However, these can be difficult to quantify and depend on the whistleblower’s social norms, moral standards, and cultural environment. Social and moral incentives come under the broader category of ethical behavior and more stringent whistleblowing laws. Hence, this work argues that financial incentives can be more prominently and easily applied. However, as indicated by Berger et al. (2017), when whistleblowers focus on financial incentives, they tend to delay the revelation of wrongdoing until it results in significant losses. In this context, whistleblowers see revelation as an economic decision rather than an ethical one (Berger et al. 2017; Brown et al. 2016; Latan et al. 2019c), and this action is therefore included in the category of prosocial behavior. However, external whistleblowers often recognize that financial incentives play an important role in their decision to act. Given that there are several financial incentive programs in place outside organizations, compared with the relative rarity of internal incentive programs, this motivates external whistleblowers. The results of previous studies by Andon et al. (2018), Latan et al. (2019c), Lee et al. (2020) and Rose et al. (2018) show that financial incentives have a positive effect on whistleblowing intention. Based on the above discussion, our second hypothesis is:

#### H2

Financial incentives have a positive effect on online whistleblowing intention.

### Opportunity to Blow the Whistle

This work defines opportunity as the availability of resources to support observers in revealing wrongdoing. Several factors increase opportunities for external whistleblowers: the availability of open reporting channels; support from bystanders; support from family and friends; as well as moral values and ethical standards. In addition, information technology also plays an important role in online whistleblowing intention. As Lam and Harcourt (2019) argue, the use of online channels for whistleblowing makes it possible to share information widely through messages, photographs and videos, with speed and anonymity. In addition, support from social media or website providers, technology (hardware and software) and the general public provide further opportunities for online whistleblowing. Several scholars even analogize such opportunities for disclosure as procedural justice (Brennan and Kelly 2007; Seifert et al. 2014; Soni et al. 2015); that is, organizational justice relating to procedures in the workplace. When the general climate of procedural justice is elevated, observers may choose not to remain silent when faced with wrongdoing.

Opportunity also relates to the type of wrongdoing and the individual whistleblower, which may require different reporting channels. For example, where the fraud takes place online, disclosure of the wrongdoing also tends to use online platforms. In addition, external whistleblowers may be forced to choose online channels to report wrongdoing as opposed to internal channels, because they do not have internal access to the organization. As theorized by Smaili and Arroyo (2019), additional opportunities increase potential whistleblowers’ intention to speak out about wrongdoing. Research from Brown et al. (2016) and Latan et al. (2019c) indicates that opportunities have a positive effect on the intention of accountants to reveal wrongdoing. Based on the above discussion, the third hypothesis derived is:

#### H3

Opportunity has a positive effect on online whistleblowing intention.

### Rationalization of Whistleblowing

Smaili and Arroyo (2019) define rationalization as a process of cognitive justification underlying the decision to blow the whistle. This represents a process of reasoning undertaken by whistleblowers considering their action (or inaction) when faced with wrongdoing, culminating in a decision which is in line with their own moral standards (Brown et al. 2016; Latan et al. 2019c). Rationalization is a cognitive process that enables observers to distinguish, for instance, between what actually happened and what should have happened (MacGregor and Stuebs 2014). Near and Miceli (2011) illustrate this process as a mechanism by which observers consider whether action should be taken to help victims. For observers with higher ethical standards, the process of rationalization may not be difficult, because they can easily make a decision and determine whether an instance of wrongdoing was serious, illegal or immoral before blowing the whistle. However, for observers with lower ethical standards, the rationalization process may not progress as smoothly, as they tend to be less engaged and more afraid of reporting wrongdoing. In this situation, the observer does not want to take any risks and therefore may remain silent (Reckers-Sauciuc and Lowe 2010).

A rationalization process is necessary before a decision to blow the whistle is made. This process usually aligns with the observer’s beliefs regarding wrongdoing and moral standards. Several previous studies have found that the rationalization process has a positive effect on helping the whistleblower make the decision to act (Brown et al. 2016; Latan et al. 2018, 2019b; MacGregor and Stuebs 2014; Rehg et al. 2008). Based on the above discussion, the fourth hypothesis is:

#### H4

Rationalization has a positive effect on online whistleblowing intention.

### Whistleblowing Capability

Capability relates to the whistleblower’s ability to deal with wrongdoing. Capability relates to the individual whistleblower’s strength, which can be considered a panacea when engaging with wrongdoing. Wolfe and Hermanson (2004) argue that capability is an important element of the fraud diamond model, because it involves psychological and technical factors that help the observer to speak out. The characteristics of whistleblowing capability include: being in the right position to blow the whistle; having the confidence to expose and report wrongdoing; having adequate technological skills; and having the ability to take action while under threat of retaliation. The capability element is also related to a proactive personality in whistleblowers, because, in general, those with a proactive personality feel more comfortable taking action regarding issues in the workplace (Miceli et al. 2012).

In relation to online whistleblowing intention, such capabilities help the observer because online platforms require a certain level of ability to operate. A whistleblower with high capability will therefore be able to report wrongdoing more easily through such platforms. That is, they will not experience the fear of retaliation and threats that come along with more traditional methods. Conversely, an observer with lower capabilities may be reluctant to report wrongdoing and therefore choose to remain silent. Based on previous studies conducted by Boyle et al. (2015) and Wolfe and Hermanson (2004), whistleblowers’ capabilities do assist them in reporting wrongdoing. Hence, it seems that capability has a positive effect on online whistleblowing intention. Based on the above discussion, the fifth hypothesis is:

#### H5

Capability has a positive effect on online whistleblowing intention.

**Fig. 1:**
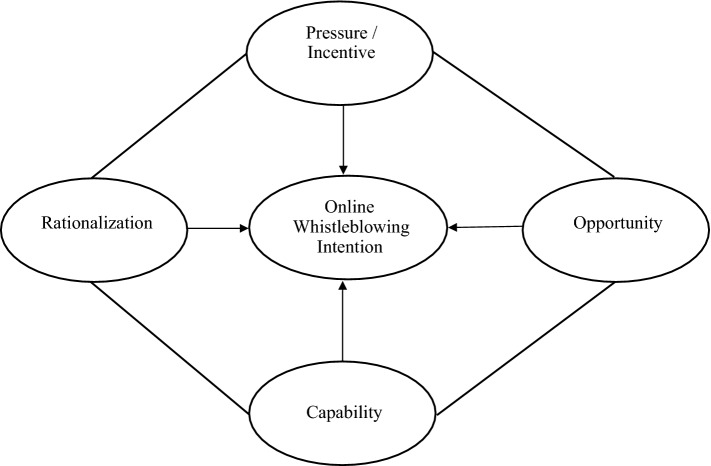
Theoretical framework for understanding online whistleblowing intention

Figure 1 portrays the research framework empirically tested in this work.

## Research Method

### Sample and Data Collection

The sample used in this study consists of customers who shop using Indonesian online stores. Customers were chosen as the sample in this study because, based on the ACFE report from 2018, they are particularly active subjects in observing and revealing wrongdoing, with the highest percentage after employees. In addition, based on the aforementioned ACFE report (2018), the retail sector, including online stores, experiences a high rate of fraud, due to the recent increase in food fraud and wrongful business practices (Moy 2018); however, there is a lack of existing research addressing this area. Since the overall sampling frame in our case is unknown, with the total number of customers being difficult to identify, it is not possible to apply the use of probability sampling in this study to obtain a random and representative sample. Therefore, we have used non-probability sampling to collect data through online surveys. The use of non-probability sampling is considered appropriate when the number of respondents is very large and uncountable. Customers were identified using snowball sampling, through social media and ratings given on online stores’ websites. In total, 1069 online shopping customers throughout Indonesia agreed to participate in the survey.

A questionnaire link was sent to each customer, after pre-testing to minimize potential bias that might have threatened the validity of the results. This included accounting for possible measurement errors and identifying sources of bias in the survey method (e.g., non-response bias, common method bias, social desirability bias) to improve the quality of the survey (Podsakoff et al. 2012; Spekle and Widener 2018) and ensure the questionnaire was understood by the customers (Fowler 2013). This process involved asking three senior academics for their opinions and suggestions to assess the content validity of the questionnaire (Rossiter 2011), leading to improved clarity. In addition, the questionnaire was originally composed in English, and a back-translation procedure—from English to Indonesian and back into English—was used to ensure clarity of content. The final version of the questionnaire was first sent to 48 customers in order to conduct preliminary data analysis, assessing the validity and reliability of the indicators to ensure the feasibility of the survey instrument. The pre-testing results indicated that the questionnaire had good validity and reliability, making it suitable for further use.

Data were collected during the period October 2018–March 2019, with a total number of 1069 questionnaires sent out. The questionnaire was sent via e-mail and social media and followed up with a notification message to ensure that the questionnaire was received. This method is considered the best way of reaching a broad field of respondents at low cost and in a short time-frame (Dillman et al. 2014). In order to increase the response rate, a reminder e-mail/message was sent at the end of each month and several telephone calls were made to those customers who had only provided telephone numbers and had not yet responded. In addition, customers were assured of their anonymity and that their names and details would not be disclosed. Informed consent was considered to have been obtained when customers completed the survey and sent it back to us, as in the conclusion of the survey they agreed that their responses would be used in this study. Finally, for the purpose of testing non-response bias (Dillman et al. 2014; Fowler 2013), the time span of data collection was set at five months.

In total, 244 questionnaires were returned. From this initial rate of return, 37 were excluded due to being incomplete, giving a final response rate of 19.36%. Following Baruch and Holtom (2008), a response rate of  > 15% is widely considered acceptable among studies using the survey method. Groves et al. (2009) argue that online surveys tend to produce low response rates, but that the results are not jeopardized by bias as a result of this, as long as there is no significant difference between the samples of respondents and non-respondents. In order to ensure that the results were free from non-response bias, early and late responders were tested and compared, with the assumption that the late responders represent customers who did not respond to the survey (Fulton 2018). While sometimes questioned, this approach has been widely used in social sciences research. Groves (2006) suggests using a post hoc test as a more robust approach to detect this bias. Hence, both approaches were used in this study to test for non-response bias. First, a t-test was run to assess differences in the means of the two sample groups. The results did not show any significant differences between early and late responders. Table 1 shows the results for Levene’s test, which was significant at a value of  > 0.05, indicating that the assumption of homogeneity variance was fulfilled. Furthermore, significance values  > 0.05 for equality of means were obtained in both sample groups for the variables tested. These results indicate that non-response bias is not currently detected in our data. However, we cannot confirm that our set of respondents is identical with the set of non-respondents, because this type of sample cannot be generalized in this way. Second, no differences in socio-demographic variables were found when running a Bonferroni test. This result indicates that the response rate is similar across subgroups, which means that non-response bias was not found in this case. However, we acknowledge that non-response bias may still exist, despite the fact that our testing did not detect it.

**Table 1:** Assessment of non-response bias and social desirability bias

Construct	Sig. Levene’s test	Sig. *t*-test for equality of means	Social desirability bias
Pressure (PRS)	0.661	0.786	–
Financial incentive (FNI)	0.552	0.870	–
Opportunity (OPR)	0.554	0.267	–
Rationalization (RNL)	0.367	0.214	–
Capability (CPB)	0.972	0.278	–
Online whistleblowing (OWB)	0.595	0.176	0.247

Furthermore, the results were assessed for other biases, such as Common Method Bias (CMB), which often arises when using the survey method (Podsakoff et al. 2012). A full collinearity VIF (AFVIF) was used, an approach proposed by Kock (2017) to assess CMB by assessing the correlations between two measurements. The analysis results obtained an AFVIF value of 2.99 < 3.3, which indicates that CMB is not a threat to the results. Finally, Social Desirability Bias (SDB) was considered, a common bias which is often ignored in survey research. SDB generally refers to respondents’ tendency to select responses that reflect societally approved behavior (Nunnally and Bernstein 1994; Chung and Monroe 2003). That is, respondents tend to choose answers which reflect positively on them. In order to detect this bias, an indirect questioning approach was applied by adding additional measurement items during the initial data collection (Fisher 1993). This bias was controlled for in the context of online whistleblowing intention, and the results showed that there were no significant differences (*p* < 0.05), between the two measurements. This indicates that the target respondents did not provide different answers when taking a personal point of view compared with a third-person perspective (see Table 1). However, once again, we acknowledge that these biases may still exist, even though we did not detect them at this time. Although we have tested and controlled for both biases, we cannot fully guarantee that our data are free from these issues. A summary of respondent profiles can be seen in Table 2.

**Table 2:** Characteristics of the sample

Demographic variable	Frequency (*f*)	Percentage (%)
Gender		
Male	84	40.58
Female	123	59.42
Age (years)		
21–30	79	38.16
31–40	92	44.44
41–50	27	13.05
51–60	9	4.35
Shopping experience		
1–2 years	37	17.87
3–4 years	59	28.50
5–6 years	98	47.35
Over 6 years	13	6.28
Academic qualifications (level of education)		
High school	32	15.46
Diploma	29	14.01
Bachelor’s degree	91	43.96
Master’s degree	55	26.57
Online stores utilized		
Lazada	42	20.29
Tokopedia	58	28.02
BliBli	36	17.39
JD Indonesia	21	10.14
Shopee	14	6.76
Bukalapak	36	17.36

### Measurement Items and Scales

Measurement items and scale are core parts of quantitative research and often have effects on research results. A good measurement item must be able to capture the concept of the measured construct. This research adopts measurement items that were developed in previous whistleblowing studies. Proxies from prior studies are also used to develop several items in this study. Although our topic is a recently developed concept and few studies have so far addressed this issue (Smaili and Arroyo 2019), measurement items for constructs in this model have been established in two previous works through a series of tests and results (Brown et al. 2016; Latan et al. 2019c). We argue that these items have good validity and reliability, as well as the proven ability to measure empirically tested constructs. Hence, these items were adapted for use in the current research context with little modification. It is worth noting that using established measurement items is generally considered better practice than developing new ones, given the complexity of scale development (Fowler 2013; DeVellis 2017).

To measure the elements of the whistleblowing diamond, measurement items adapted from Brown et al. (2016), Latan et al. (2019c) and Murphy and Free (2016) were used. Specifically, the elements were divided into the following categories: PRS, FNI, OPR, RNL and CPB. First, pressure (PRS) to engage in blowing the whistle was measured using 4 items adapted from Latan et al. (2019c) and Murphy and Free (2016), with modification. We used a 7-point Likert scale ranging from 1 = “not likely” to 7 = “very likely” to measure this variable. For instance, respondents were asked “how likely are you to engage in blowing the whistle, because of the social pressure to do the right thing based on a certain situation in a scenario” and so on. Second, we measured the variable of financial incentive (FNI) using 2 items adopted from Latan et al. (2019c) and Brown et al. (2016). Once again, a 7-point Likert scale was employed, with a scale ranging from 1 = “not likely” to 7 = “very likely” to measure this variable. In the same vein, respondents were asked, for example, “how likely are you to engage in blowing the whistle, in order to gain financial incentive and reputation”. Third, the opportunity (OPR) for engaging with QRPs was measured using 4 items adapted from Latan et al. (2019c) and Brown et al. (2016). We again used a 7-point Likert scale from 1 = “not likely” to 7 = “very likely”. For example, respondents were asked about “possibilities to use online channels because of difficulties faced in the process of reporting internally” and so on. Fourth, we measured rationalization (RNL) using 5 items adopted from Latan et al. (2019c) and Murphy and Free (2016). We used a 7-point Likert scale ranging from 1 = “not likely” to 7 = “very likely” and respondents were asked questions such as “how likely are you to engage in blowing the whistle, in order to help someone else by disclosing wrongdoing”. Fifth, we measured capability (CPB) based on proxies provided by Wolfe and Hermanson (2004). A 7-point Likert scale was also used to measure this construct, this time with 5 indicators. This scale ranged from 1 = “not likely” to 7 = “very likely”. Respondents were asked questions such as “how likely are you to engage in blowing the whistle because of being in a good position to speak out” and so on. Finally, to measure online whistleblowing intention (OWB), measurement items based on studies from Lam and Harcourt (2019) were developed. This construct relates to the use of an online platform to act when observing wrongdoing, with a total of 5 items. To the best of our knowledge, measurement items for use in measuring OWB have not previously been developed. Again, we used a 7-point Likert scale with a scale ranging from 1 = “not at all” to 7 = “very much”. Respondents were asked to indicate their potential use of online reporting channels to blow the whistle based on a particular scenario. All constructs can be considered to be captured appropriately when measurement items are able to reflect what they want to measure, which is indicated by good validity and reliability.

The measurement objectives of the constructs in this model were achieved using a hypothetical scenario, with customers as actors. The scenario used in this study appears in Appendix 1. In this scenario, customers were asked to position themselves as a witness to food fraud, which is related to impaired products and wrongful business practices. We designed this scenario to capture the essence of each construct. A hypothetical scenario is the most common form of whistleblowing survey research, and explains customers’ self-reported actions in response to observed wrongdoing in certain situations (Olsen 2014). A hypothetical scenario approach was chosen because it is difficult to directly measure observation of wrongdoing in the workplace. Scenario approaches are widely used in the whistleblowing literature (Alleyne et al. 2019; Latan et al. 2019a; Park and Lewis 2019; Valentine and Godkin 2019). In addition, the use of hypothetical scenarios does possess certain limitations, because the variables are measured without real-life decisions having to be made, which in some cases may not align with reality. Nevertheless, this is currently the best way to test online whistleblowing intention.

### Data Analysis

Structural Equation Modeling (SEM), which is considered a second-generation analysis method, was employed to test our model and hypotheses. SEM has become a core part of quantitative analysis, which includes a variety of methods. The component-based SEM method, or ‘soft modeling’, was used in this study through a partial least squares path modeling (PLS-PM) approach (Hair et al. 2017; Lohmöller 1989). PLS-PM was chosen by considering a number of advantages related to its characteristics, which are superior to other SEM approaches (Latan and Noonan 2017).

PLS was initially developed for two reasons. First, to test primitive models where there is a relative scarcity of theory and knowledge (Noonan and Wold 1986). Given that this model is still primitive, due to its recent development and relative scarcity in the literature, PLS was seen as a suitable approach in this regard (Wold 1989). In addition, PLS provides a high level of predictive accuracy in terms of model estimation and balancing causal-predictive relationships between variables (Lohmöller 1989; Rigdon 2013). Second, PLS relaxes the heavy assumptions arising from the covariance-based SEM (CB-SEM) approach. That is, PLS employs soft modeling with light assumptions, because it is based on linear aggregates and offers flexibility for various applications in real-world cases (Sellin 1988). One advantage of PLS is that it avoids Heywood cases and factor indeterminacy, which can occur in CB-SEM, using the principle of consistency at large. Finally, PLS-PM provides user-friendly software with a graphical user interface. In this case, PLS offers advanced features that make it easy to run without the need to use syntax codes.

Given the long journey of PLS towards achieving popularity in social sciences research, as well as the currently available guidelines and standards for reporting the results of PLS analysis, we followed the step-by-step guidelines for best practice which are available in the literature (Benitez et al. 2020; Hair et al. 2019; Latan 2018) in reporting our PLS analysis results. Before analyzing our model, we calculated the adequacy of the sample size for our parameter estimates. We used the gamma-exponential method, and found that the minimum sample size for our model was 146 cases (where the minimum absolute significant path coefficient = 1.97, significant level = 0.05 and required power level = 0.80), which our study meets.

In short, we used a three-step approach to report the results of our PLS analysis as follows. First, we report the results of the outer model, which is related to the assessment of the measurement model, to show that the indicators in the model are valid and reliable. Second, we report the results of the inner model, which is related to the assessment of the structural model, by looking at standard metrics in PLS and testing our hypotheses. Finally, we will provide the results of several robustness tests which were conducted to ensure that our main analysis results were free of certain systematic biases. We used the SmartPLS 3 software to analyze our data (Ringle et al. 2015). We implemented a number of specific settings before running this software. In the PLS algorithm settings, we selected the path weighting scheme with the maximum number of iterations set at 300 and a stop criterion of 10^−7^ (= 1.0E−07). In terms of bootstrapping, we used 5000 subsamples to obtain stability of model estimates through confidence interval methods, namely a bias-corrected and accelerated (BCa) bootstrap. In addition, we set the level of significance to reject the null hypothesis at 5% (one-tailed). The results obtained are described below.

## Results

Before reporting the results of our main analysis, we conducted factor analysis using principal component analysis (PCA) to assess the unidimensionality of construct measurements in our model. We obtained Kaiser–Meyer–Olkin Measure of Sampling Adequacy (KMO-MSA) values of  > 0.5 for each construct in our model and rotation of matrix component values of  > 0.60 for all items (see Table 3). From this, we can conclude that the measurement items form a single factor for each construct, and the items we developed (in this case the CPB and OWB) have good unidimensionality. Furthermore, we obtained the following main analysis results, which were extracted from the SmartPLS output.

**Table 3:** Measurement model assessment of diamond elements

Indicator/item	Code	PCA	Mean	SD	FL	AVE	*α*	*ρ*_A_
(A) Pressure (PRS)						0.592	0.768	0.778
Social pressure to do the right thing	PRS1	0.784	5.357	1.062	0.792			
My sense of moral obligation to report wrongdoing	PRS2	0.809	5.348	1.070	0.821			
My religion leading me to do the right thing	PRS3	0.809	4.899	1.294	0.795			
My sense of duty to report wrongdoing	PRS4	0.667	4.734	1.504	0.660			
(B) Financial Incentive (FNI)						0.825	0.789	0.795
Standing to gain financially by reporting wrongdoing	FNI1	0.909	4.686	1.436	0.898			
Standing to gain in reputation by reporting wrongdoing	FNI2	0.909	4.720	1.146	0.919			
(C) Opportunity (OPR)						0.734	0.879	0.879
The firm hinders (or ignores) reporting	OPR1	0.857	5.092	1.128	0.860			
Difficulties faced in the process of internal reporting	OPR2	0.875	4.754	1.225	0.872			
Internal reporting is likely to be ineffective in ending the wrongdoing	OPR3	0.862	4.710	1.213	0.860			
Potential for retaliation by the firm	OPR4	0.833	5.159	1.292	0.836			
(D) Rationalization (RNL)						0.672	0.878	0.878
Helping the victims of the situation	RNL1	0.831	5.512	1.150	0.829			
Helping someone else by disclosing wrongdoing	RNL2	0.873	5.343	1.217	0.862			
Did not consider whether the action was right or wrong at the time	RNL3	0.774	4.744	1.437	0.783			
Did not consider the consequences of this action	RNL4	0.813	4.976	1.181	0.814			
Did not think this action was so bad	RNL5	0.806	5.498	1.137	0.809			
(E) Capability (CPB)						0.675	0.879	0.881
Being in a good position to speak out	CPB1	0.749	4.802	1.131	0.754			
Having the confidence to disclose it	CPB2	0.848	4.981	1.333	0.844			
Having the relevant technological skills	CPB3	0.817	5.174	1.393	0.813			
Mental ability to think effectively about speaking out	CPB4	0.846	4.952	1.487	0.845			
Immunity to retaliation	CPB5	0.842	5.213	1.245	0.847			

*PCA* principal component analysis, *FL* factor loading, *SD* standard deviation, *AVE *average variance extracted, *α* Cronbach’s Alpha, *ρ*_*A*_ Dijkstra–Henseler’s rho_A

### Measurement Model Assessment

**Fig. 2:**
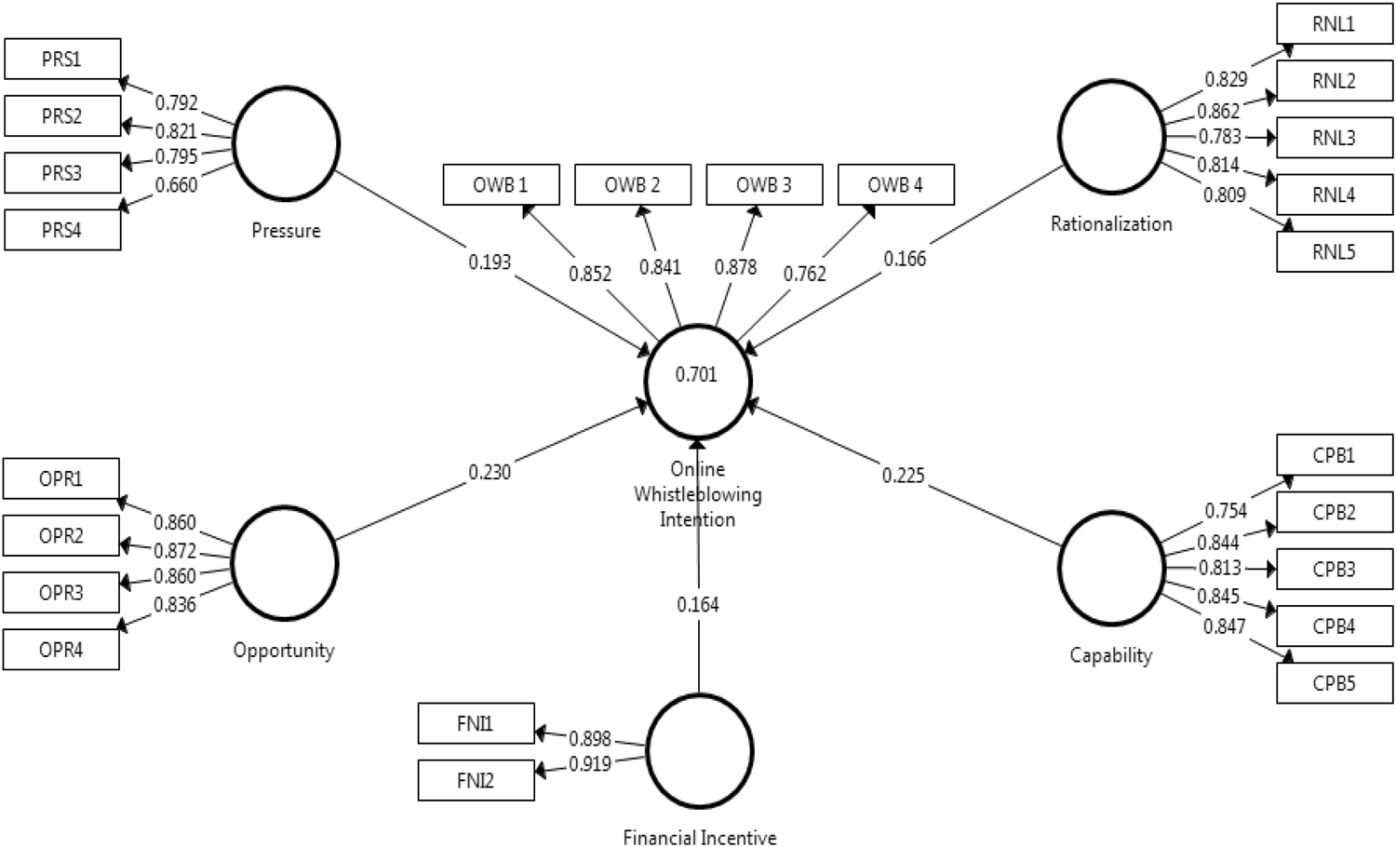
Evaluation of measurement and structural models

We depended on several core metrics that are commonly used in PLS to test convergent and discriminant validity, as well as internal consistency reliability. First, we inferred convergent validity through loading factors and average variance extracted (AVE). The recommended values for the loading factor of the indicators in the model should be  > 0.708, and the AVE value, used to explain the construct variance, should be  > 0.5 (Benitez et al. 2020; Hair et al. 2019; Latan 2018). However, in many cases, a loading factor value between 0.50 and 0.60 is obtained, due to the large number of items in the model. Such a value can still be acceptable, as long as the AVE value meets the threshold required to strengthen content validity. In Tables 3 and 4 we depict the results of our analysis for convergent validity. Our results fulfilled the rule of thumb and the threshold values for good convergent validity (see Fig. 2). Furthermore, we assessed construct reliability in the model using two measures: Cronbach’s alpha (α) and Dijkstra-Henseler’s *ρ*_A_. Cronbach’s alpha is a conservative measure and indicates the lower bound of reliability. This measure is useful when a small sample size is combined with a low number of indicators, while *ρ*_A_ serves as a good representation of a construct’s reliability (Nunnally and Bernstein 1994). The recommended threshold values for Cronbach’s alpha (α) and *ρ*_A_ range from 0.80 to 0.90. The results of our analysis, presented in Tables 3 and 4, show that the construct reliability in the model fulfills this rule of thumb.

**Table 4:** Measurement model assessment of online whistleblowing intention

Indicator/Item	Code	PCA	Mean	SD	FL	AVE	*α*	*ρ*_A_
(F) Online Whistleblowing (OWB)						0.696	0.854	0.862
Reporting through social media channels of related authorities (e.g., Facebook, Twitter etc.)	OWB1	0.850	5.319	1.190	0.852			
Using online publishing organizations to make information known to the relevant authorities (e.g., WikiLeaks)	OWB2	0.845	4.647	1.230	0.841			
Reporting through online platforms provided by the related authorities (e.g., E-mail, Online Application, etc.)	OWB3	0.871	4.874	1.298	0.878			
Using personal online media sites (e.g., blogs, websites or YouTube) to disclose information to the relevant authorities	OWB4	0.767	4.928	1.142	0.762			

*PCA*  principal component analysis, *FL* factor loading, *SD* standard deviation, *AVE* average variance extracted, *α* cronbach’s Alpha, *ρ*_*A*_ Dijkstra–Henseler’s rho_A

In addition to assessing convergent validity, we also assessed discriminant validity to ensure that the measurements of separate constructs are not unduly correlated with each other. We used the Heterotrait-Monotrait (HTMT) criterion, a new approach developed in PLS-PM to assess discriminant validity. The rule of thumb for assessing discriminant validity is indicated by a threshold value of  > 0.90 for HTMT, which indicates conceptually similar constructs, while HTMT values < 0.85 indicate conceptually different constructs (Lucianetti et al. 2018; Seles et al. 2019). From our results, shown in Table 5, we can conclude that the HTMT value is significantly lower than the specified threshold value. Hence, discriminant validity is fulfilled for our measurements. This means that each construct measurement in the model measures a different concept and the measurements are not correlated with each other.

**Table 5:** Assessment of discriminant validity using HTMT

Construct	1	2	3	4	5	6
CPB	**(0.900)**					
FNI	0.672 [0.584;756]	**(0.900)**				
OWB	0.835 [0.773;893]	0.746 [0.643;843]	**(0.900)**			
OPR	0.793 [0.710;871]	0.668 [0.557;766]	0.771 [0.691;840]	**(0.900)**		
PRS	0.827 [0.752;896]	0.763 [0.662;856]	0.610 [0.538;778]	0.765 [0.617;813]	**(0.900)**	
RNL	0.811 [0.746;869]	0.598 [0.478;707]	0.825 [0.752;888]	0.554 [0.401;703]	0.559 [0.493;622]	**(0.900)**

Brackets show the lower and upper bounds of the 95% BCa confidence intervals. The diagonal lines indicate the cut-off values for HTMT

### Structural Model Assessment

We used the inner model of PLS to assess the structural model in relation to the quality of the PLS model; this allowed us to assess the variance in the model which can be explained, the magnitude of the influence and contribution of each variable and the significance of the relationships between the hypothesized variables. We used core metrics to assess the structural model, as recommended by several scholars (Benitez et al. 2020; Hair et al. 2019; Latan 2018). This consists of coefficient of determination (*R*^2^), effect size (*f*^2^), predictive relevance (*Q*^2^) and variance inflation factor (VIF). In addition, we assessed our model’s out-of-sample predictive power by implementing the PLS predict procedure (Dolce et al. 2017).

The results of the structural model evaluation we obtained are depicted in Table 6. As shown in Table 6, we obtained *R*^2^ and adj. *R*^2^ values for OWB of 0.694 and 0.701, respectively, which indicates the percentage of variance which can be explained by the predictors in our model. As Hair et al. (2019) note, these values fall into the large category; however, values that are too high, for example > 0.90, indicate over-fit and the occurrence of collinearity between variables. However, the magnitude of these values will depend on the number of predictor variables in the model, in relation to complexity and sample size. In the field of business ethics for instance, both values are often found to be lower than 0.50, considering the broad scope and complex phenomena used to explain the relationships between variables.

**Table 6:** Structural model assessment

Construct	*R*^2^	Adj. *R*^2^	*f* ^2^	*Q*^2^	VIF	AFVIF
Pressure (PRS)	–	–	0.038	–	3.252	–
Financial incentive (FNI)	–	–	0.054	–	1.662	–
Opportunity (OPR)	–	–	0.050	–	2.838	–
Rationalization (RNL)	–	–	0.033	–	2.838	–
Capability (CPB)	–	–	0.067	–	2.527	–
Online whistleblowing (OWB)	0.701	0.694	–	0.449	–	2.939

In addition, we obtained effect size values (*f*^2^) produced by the predictors in our model which ranged from 0.033 to 0.067, falling into the medium category. These values define the contribution of each predictor in the model to explain the variance of the dependent variable (in our case, OWB). The greater the *f*^2^ value, the more important the role of this predictor variable in the model. Conversely, a smaller *f*^2^ value indicates a relationship between predictor and outcome that is not significant; therefore, no variance in the model is explained. We also assessed predictive relevance (*Q*^2^) as an alternative measure of R^2^ to show the predictive power of our PLS model. A *Q*^2^ value larger than zero is meaningful and indicates that the PLS model is worth testing. We ran a blindfolding procedure with omission distance (*D*) = 7 and produced a *Q*^2^ value of 0.499, indicating the large predictive relevance of our PLS model. In addition, we obtained VIF values for each predictor in the model of less than 3.3, which indicates no significant correlation or collinearity between predictor variables in the model.

Finally, we tested the model’s out-of-sample predictive power by running the PLS predict algorithm (Dolce et al. 2017) to generate holdout sample-based point predictions for the constructs in our model. Given that our sample size meets the minimum requirements and is large enough, we used ten folds and ten repetitions, and compared the root mean squared error (RMSE) values from the PLS-PM analysis with those generated by a naïve linear benchmark (Dolce et al. 2017; Hair et al. 2019). The results indicate that the PLS-PM analysis yields lower prediction errors than the naïve benchmark for most of the indicators related to PRS, FNI, OPR, RNL, CPB and OWB, offering clear support for our model’s predictive power. In addition, $Q_{{{\text{predict}}}}^{2}$ values > 0 indicate that our model outperforms the naïve benchmark (i.e., the indicator means from the analysis sample).

### Testing of Hypotheses

We tested the derived hypotheses for the relationships between variables by performing a bootstrapping procedure. In testing these hypotheses, we assessed the direction of the path coefficients, and accepted or rejected each hypothesis based on a 95% confidence interval (CI), generated at the 5% significance level (one-tailed). Overall, our results support the hypotheses on the relationships between predictors and outcome. As shown in Table 7, we found that the relationships between PRS → OWB, FNI → OWB and OPR → AWB were significant, with beta (β) values of 0.193, 0.164 and 0.230, respectively, and significance at *p* ≤ 0.05 at 95% CI. From these results we can conclude that H1, H2 and H3 are fully supported. Additionally, we found the relationships RNL → OWB and CPB → OWB to be significant, with beta (β) values of 0.166 and 0.255, respectively, and significance at *p *≤ 0.05 at 95% CI. Hence, we can conclude that H4 and H5 are also fully supported.

**Table 7:** Testing of hypotheses

Structural path	Coef (β)	SD	*p* value	95% BCa CI	Conclusion
PRS $\to$ OWB	0.193	0.066	0.002**	(0.295, 0.075)*	H1 supported
FNI $\to$ OWB	0.164	0.059	0.003**	(0.261, 0.066)*	H2 supported
OPR $\to$ OWB	0.230	0.094	0.007**	(0.387, 0.079)*	H3 supported
RNL $\to$ OWB	0.166	0.066	0.006**	(0.277, 0.058)*	H4 supported
CPB $\to$ OWB	0.225	0.093	0.008**	(0.389, 0.082)*	H5 supported

**,*Statistically significant at the 1 percent and 5 percent levels, respectively

### Robustness Tests

We performed several robustness tests to ensure that our main results are free from certain biases, such as endogeneity, non-linearity and unobserved heterogeneity. Several scholars (Peel 2018; Zaefarian et al. 2017) have noted these biases as a threat to results that can lead to mistakes in drawing conclusions, and therefore need to be tested. First, we tested endogeneity bias to assess whether there were interventions from omitted variables, the presence of reverse causality relationships, or other potential errors (e.g., sample-selection bias). To ensure that this bias did not affect our results, we conducted the Heckman test using a two-step procedure with the help of the Stata software. In the first step, we ran our model and data without controlling for endogeneity bias. In the second step, we controlled for the effects of endogeneity bias by including a third variable in our model equation. Our results, shown in Table 8, indicate that there are no differences in results whether or not this bias is controlled for, which indicates that endogeneity bias does not occur in our data or model.

**Table 8:** Assessment of endogeneity bias using the Heckman test

Test	Coef (β)	*p* value	*z*	Conclusion
PRS $\to$ OWB (Selection DV = CPB; IV = FNI, OPR)	0.784	0.000**	15.38**	No bias present
FNI $\to$ OWB (Selection DV = RNL; IV = PRS, OPR)	0.495	0.000**	11.00**	No bias present
OPR $\to$ OWB (Selection DV = PRS; IV = FNI, RNL)	0.736	0.000**	16.57**	No bias present
RNL $\to$ OWB (Selection DV = OPR; IV = PRS, FNI)	0.579	0.000**	14.75**	No bias present
CPB $\to$ OWB (Selection DV = FNI; IV = OPR, RNL)	0.541	0.000**	15.07**	No bias present

*DV* dependent variables, *IV* is independent variables

**,*Statistically significant at the 1 percent and 5 percent levels, respectively

Second, we examined whether non-linear effects occur in the relationships between variables in our model, to ensure that linear assumptions are fulfilled. When a non-linear effect appears and there is assumed to be a linear relationship, this indicates a mirage. We tested this effect by using Ramsey’s regression specification error test (RESET) and quadratic functions in the SmartPLS software. The results of our analysis for this bias, presented in Table 9, fully support a linear relationship between variables in the model. We found the presence of non-linear relationships between variables to be insignificant, with *f*^2^ falling in the small category and *p* value > 0.05 for Ramsey’s RESET. This indicates that non-linear effects do not appear in our model (Wooldridge 2020).

**Table 9:** Assessment of non-linear effects

Structural path	Coef (β)	*p* value	*f*^2^	Ramsey’s RESET
PRS * PRS $\to$ OWB	− 0.035	0.311^n.s^	0.005	
FNI * FNI $\to$ OWB	0.058	0.056^n.s^	0.014	
OPR * OPR $\to$ OWB	− 0.071	0.194^n.s^	0.019	*F* (1.835) = 0.74, *p* = 0.497
RNL * RNL $\to$ OWB	− 0.102	0.139^n.s^	0.045	
CPB * CPB $\to$ OWB	0.076	0.198^n.s^	0.023	

^n.s^is not significant

Finally, we examined unobserved heterogeneity bias, which usually arises from differences between segments or clusters of the target population. Scholars usually assume that data come from a single population, but under certain conditions it may not. Hence, this bias usually occurs when performing sample selection. To test for this bias, we ran Finite Mixture PLS (FIMIX-PLS). After assessing goodness of fit and performing multiple procedures, such as Akaike’s information criterion (AIC_3_) and consistent AIC (CAIC), we found that FIMIX-PLS gave a final result of *k* = 1, indicating that our data is free from this bias.

## Discussion and Implications for Theory and Practice

The intention to blow the whistle through online channels such as social media and other online platforms has become an area of study demanding urgent attention at this time (Cherry 2012; Bosua et al. 2014). The present research attempts to fill this gap by expanding the concept of the whistleblowing triangle, adding to it the fourth element of the fraud diamond—capability—and testing the expanded concept, called the whistleblowing diamond, as a predictor of online whistleblowing intention, using a sample of customers in Indonesia. Our findings answer the research calls of Vandekerckhove et al. (2014a) and Lam and Harcourt (2019) to provide the first empirical evidence related to these contemporary methods of blowing the whistle. In general, we find empirical support for the whistleblowing diamond elements in relation to online whistleblowing intention in Indonesia.

Specifically, our main contributions can be presented as follows. First, we have identified a positive and significant effect on the relationship between pressure and online whistleblowing intention, where PRS encourages individual intention to blow the whistle. Our findings imply that whistleblowers are motivated by social pressure to make the decision to report wrongdoing. Whistleblowers who react upon discovering wrongdoing by an organization may be bound by moral values or religious loyalty. In the Indonesian environment, such values are highly emphasized. On the other hand, they may report wrongdoing because of the level of damage and loss caused by the wrongdoing, in which case the decision to report takes into account the possibility of helping the victims. Therefore, the whistleblower is under social pressure and is motivated by human relations to blow the whistle. Our findings support the propositions of Smaili and Arroyo (2019) and empirical evidence from Brown et al. (2016), Chen and Lai (2014) and Latan et al. (2019c), which indicate that PRS has a positive effect on OWB.

Second, we identified a positive and significant effect on the relationship between financial incentives and online whistleblowing intention. Financial incentives are compensation programs or rewards given to whistleblowers who report serious wrongdoing which has the potential to cause significant losses. As pointed out by several scholars (Andon et al. 2018; Berger et al. 2017; Latan et al. 2019c), the expectation of gaining financial incentives is another driving force for whistleblowers to report wrongdoing. This economic motive is a prosocial behavior, where in addition to helping the victims, whistleblowers also desire reward. In Indonesia, financial incentives are given for uncovering wrongdoing. Dozier and Miceli (1985) underline that such behavior is often found in various cases of whistleblowing. Several previous studies corroborate our findings (Andon et al. 2018; Latan et al. 2019c; Rose et al. 2018), where financial incentives trigger online whistleblowing intention.

Third, we found evidence of a positive relationship between opportunity and online whistleblowing intention, where OPR increased the intention to blow the whistle. Because whistleblowers are often operating under the threat and fear of retaliation, they will choose the easiest opportunity to blow the whistle. Opportunities are always related to the availability of supporting resources that help whistleblowers to take action. In addition, the availability of mobile devices allows the opportunity to blow the whistle even more easily (Lam and Harcourt 2019). Previous studies by Brown et al. (2016) and Latan et al. (2019c) show that such opportunities increase the intention to blow the whistle. That is, the easier the reporting channel is considered to be by whistleblowers—in this case, online whistleblowing—the more they will tend to blow the whistle, due to the minimized perceived level of risk. Among the sample analyzed, the use of social media seems to encourage customers to see more opportunities to disclose wrongdoing; in particular because the sample analyzed recognizes that social media may reduce the potential for retaliation by firms.

Fourth, we identified evidence of a positive relationship between rationalization and online whistleblowing intention, where RNL increased the intention to blow the whistle. Rationalization is a process of reasoning used to choose between two options that are opposed to each other. In many cases, an observer may be confused in determining their own course of action, due to the inconsistency of the results of whistleblowing. For example, there are whistleblowers who receive praise when revealing wrongdoing, while others suffer retaliation. In such situations, rationalization helps the whistleblower to make a decision. Given the conditions in Indonesia, which prioritize values, morals and ethics, the rationalization process will be effective. Consequently, this process is considered to trigger the intention of the whistleblower to report wrongdoing. Several previous studies support this finding (Brown et al. 2016; Latan et al. 2019b, c; Near and Miceli 2011), resulting in a positive relationship between RNL and OWB.

Finally, we can support a positive and significant effect on the relationship between capability and online whistleblowing intention. It is worth noting that the coef. β value in this relationship was the highest among all relationships tested, indicating that the addition of the capability element to the whistleblowing triangle was pertinent in assessing online whistleblowing intention. Given the capabilities of whistleblowers in relation to personality and their ability to speak out, this is a factor that plays an important role in whistleblowing actions. Our findings fully support the role of capabilities in OWB. Adequate capability will help the observer when reporting wrongdoing that is considered serious. Our findings support previous research that indicates a positive relationship between CPB and OWB (Boyle et al. 2015; Wolfe and Hermanson 2004). The use of virtual channels, such as social media, has a significant relationship with the capability of the sample analyzed to blow the whistle.

Our research provides a number of original theoretical and practical implications, as follows. In terms of theoretical implications, our findings add new evidence and extend the state-of-the-art research in the whistleblowing literature in complex, digitally enabled organizational contexts. More precisely, this can be considered the first empirical study to use online channels as a contemporary approach to whistleblowing. While most studies have dealt with traditional approaches to whistleblowing, such as using internal and external channels (Alleyne et al. 2018; Latan et al. 2018; Park and Blenkinsopp 2009), understanding of trends related to online whistleblowing is still limited (Bosua et al. 2014; Lam and Harcourt 2019), despite being a key contemporary issue in the field. In addition, our research contributes theoretically to the development of the whistleblowing triangle model (Smaili and Arroyo 2019; Latan et al. 2019c), by developing the model into the whistleblowing diamond.

To summarize, this article encapsulates a number of relevant implications regarding the previous literature. In line with the Theory of Planned Behavior (TPB) as applied to whistleblowing theory (Brown et al. 2016), our research results are aligned with previous theory which indicates that pressure has a positive effect on the intention to blow the whistle (Smaili and Arroyo 2019), and that internal pressure motivates potential whistleblowers to act (Chen and Lai 2014; Latan et al. 2019c). Our findings regarding the relationship between financial incentives and whistleblowing intention confirm previous theory such as Andon et al. (2018), Latan et al. (2019c), Lee and Fargher (2018) and Rose et al. (2018). We also add to Smaili and Arroyo (2019), Brown et al. (2016) and Latan et al. (2019c) because our findings indicate that, indeed, opportunities can have a positive effect on the intention of customers to reveal wrongdoing. Regarding the relationship between rationalization and online whistleblowing intention, our findings also suggest a positive link, confirming a number of prior studies (Brown et al. 2016; Latan et al. 2019b; MacGregor and Stuebs 2014; Rehg et al. 2008). We add to the developing debate suggesting that whistleblowers’ capabilities have a positive effect, assisting them in reporting wrongdoing (Boyle et al. (2015); Wolfe and Hermanson (2004).

In terms of practical implications, our findings offer the following contributions. The sample analyzed prefers to report wrongdoing by means of social media (e.g., Facebook and Twitter), rather than using other online platforms or channels such as WikiLeaks, blogs and YouTube. The primary reasons for this appear to be due to opportunity and the potential of avoiding retaliation from firms. Therefore, firms should improve their communication with customers through the use of big data analytics in order to monitor comments from customers within their online social media channels and thus identify customers’ perception of wrongdoing by firms. Firms may thereby correct themselves, explaining potential misunderstandings or misalignment of customers’ expectations and, consequently, firms may improve customers’ satisfaction and loyalty. The identification of the channels preferred by customers to disclose wrongdoing is important for firms to enhance their relationship with customers, as well as to improve the services they provide. In addition, firms can avoid problems with their image since they can proactively monitor customers’ social media interactions, as it has been identified that social media is the virtual channel most preferred by customers to report wrongdoing. Investing in big data analytics would be a better way to allocate resources, rather than investing in firms’ own online platforms for communication with customers.

## Limitations and Future Research Directions

As with all research, this study has certain inevitable limitations. First, our study only examined whistleblowing intention, without considering actual behavior. As pointed out by Bjørkelo and Bye (2014) and Culiberg and Mihelič (2017), most of the previous research in the whistleblowing literature has focused on whistleblowing intention rather than actual whistleblowing. Both factors have advantages and disadvantages: actual whistleblowing tends to be difficult to measure, while the intention to blow the whistle may be reported differently in a study compared to action taken in a genuine situation. A meta-analysis study by Mesmer-Magnus and Viswesvaran (2005) concluded that predictors of the intent to blow the whistle may differ from actual whistleblowing, in which the results were found to be stronger for intention than actual behavior. Second, our main findings may not be generalizable to other cultural contexts. As explained by Vandekerckhove et al. (2014b), research on whistleblowing requires different methods and research design in each country and society. Furthermore, the concept of whistleblowing may have different meanings in languages around the world; a cross-cultural comparison study by Patel (2003) provides preliminary evidence indicating this difference. Finally, our study only considers the diamond elements as predictors of online whistleblowing intention. In this context, we have not examined several factors, such as the nature of wrongdoing or laws and policies, as proposed by Lam and Harcourt (2019) in the framework of the online whistleblowing model.

We would suggest the following directions for future research. First, we make a research call to examine the effect of the diamond elements on actual behavior in online whistleblowing. Taking a reasoned approach to such actions, Bjørkelo and Bye (2014) suggest examining the relationship between intention and actual behavior in whistleblowing. In addition, a behavioral approach could be used to measure the actual behavior of the whistleblower. Second, the need for a cross-cultural comparison study considering the diamond elements and online whistleblowing intention should be addressed in the future. In addition, comparative studies between types of whistleblowers (online vs. external and internal) may lead to new avenues for future research (Culiberg and Mihelič 2017; Miceli et al. 2012).

## Appendix 1

### Scenario

Mia is a housewife and a graduate student from a well-known university in Indonesia. She majored in food science and nutrition. Besides being known as a smart student, she is also a critical thinker. For the past year, she has been a regular customer of food and beverage products from a company operating in Indonesia. Mia loves these products; as well as wishing to support national products, she also likes them because of the cheap price compared to competing products. She always buys these products through websites or online stores, as she is very active in using the internet and social media, and has capabilities in information systems and technology.

Everything was going well, until one day she found several irregularities in these products. In her last purchase order, Mia found that the products smelled bad, even when stored in the refrigerator. She made sure that the expiration date had not yet passed. Mia then suspected that this impaired product indicated food fraud by the company, considering she had expertise regarding this area.

To confirm her suspicions, she went to the factory where the products were manufactured. The distance was not too far, because it is still in the same province. She asked permission from the production manager to look around the factory, with the excuse of fulfilling her college assignments. In the end, she found that the products were being produced unsafely, and several other instances of fraud were also observed, such as the use of poor quality raw materials and production systems that were not environmentally friendly.

Mia then returned home and realized that food fraud and such wrongful business practices have a very serious and widespread impact. In addition to damaging the market by selling products at lower prices, which has an impact on the viability of law-abiding and honest companies, it also has potential risks to the health and nutrition of humans. The latter is a major concern, which threatens human life. This caused her to be unable to sleep for several nights.

Following these discoveries, Mia considered reporting these findings to the Consumer Protection Agency, the National Agency of Drug and Food Control or to the relevant authorities through available reporting mechanisms. She considered that if she were to reveal this misconduct through internal channels, the company might ignore it, therefore making the reporting ineffective, more difficult and not producing corrective actions. In addition, considering that she is not an employee there, Mia then decided to report this matter externally, through an online platform that she considered more appropriate. Mia wanted to forget about this case, but pressure from within herself, a sense of morality and social responsibility drove her to disclose it. However, she realized that by doing this, there were two consequences that may occur: on the one hand, obtaining financial rewards and praise; but on the other hand, being prosecuted by the company. After thinking about this for several days, Mia decided to postpone making a decision about the case until she found the right solution.
